# Knowledge of Postgraduate Dental Students on Evidence-based Dentistry and Research Methodology. An International Survey

**DOI:** 10.3290/j.ohpd.a45404

**Published:** 2020-10-13

**Authors:** Spyridon N. Papageorgiou, Despina Koletsi, Raphael Patcas, Leslie A. Will, Theodore Eliades

**Affiliations:** a Senior Teaching and Research Assistant, Clinic of Orthodontics and Pediatric Dentistry, Center of Dental Medicine, University of Zürich, Zürich, Switzerland. Study design and planning, performed experiment, collected and analysed data, wrote the manuscript, read and approved the final manuscript.; b Senior Scientist, Clinic of Orthodontics and Pediatric Dentistry, Center of Dental Medicine, University of Zürich, Zürich, Switzerland. Performed experiment, collected and analysed data, wrote the manuscript, read and approved the final manuscript.; c Senior Teaching and Research Assistant, Clinic of Orthodontics and Pediatric Dentistry, Center of Dental Medicine, University of Zürich, Zürich, Switzerland. Study design and planning, wrote the manuscript, read and approved the final manuscript.; d Professor and Director, Department of Orthodontics and Dentofacial Orthopedics, Henry M. Goldman School of Dental Medicine, Boston University, Boston, Mass. Performed experiment, collected data, wrote the manuscript, read and approved the final manuscript.; e Professor and Director, Clinic of Orthodontics and Pediatric Dentistry, Center of Dental Medicine, University of Zürich, Zürich, Switzerland. Study design and planning, wrote the manuscript, read and approved the final manuscript.

**Keywords:** clinical trial, educational assessment, epidemiologic research design, evidence-based dentistry, questionnaire, survey, systematic review

## Abstract

**Purpose:**

To assess the knowledge of postgraduate dental students about evidence-based methodology pertaining to the design, conduct, and critical appraisal of clinical trials.

**Materials and Methods:**

Senior postgraduate students were surveyed from the dental schools of three universities in Athens (Greece), Boston (USA), and Zürich (Switzerland). The proportion of students correctly answering each of the 10 questions of the survey, as well as the cumulative scores, were analysed statistically with descriptive statistics and logistic/linear regression analysis at α = 5%.

**Results:**

A total of 96 students with a mean age of 30.0 years attained an overall correct score of 45.6% ± 15.0%, with correct answers to each question ranging from 13.5% to 86.5%. The questions most frequently answered incorrectly pertained to characterising sensitivity/specificity (13.5%), the number needed to treat (14.0%), the credibility of trial synthesis in meta-analysis (23.7%), and publication bias (29.5%). The vast majority of postgraduate students could correctly identify the role of statistical power of a trial (63.8%), random allocation sequence in a randomised trial (76.0%), and blinding in a randomised trial (86.5%). Paediatric dentistry postgraduate students scored better than students from other departments (+15.1%; 95% CI: 3.0% to 27.1%; p = 0.02).

**Conclusions:**

Postgraduate students in orthodontics and other dental specialties possessed moderate knowledge on evidence-based methodology and clinical trials. Efforts should be made to integrate such subjects in university postgraduate curricula, so that future dental specialists can critically appraise such research papers.

Evidence-based oral care is receiving increasing emphasis worldwide, which means that dentists have to critically appraise a broad range of scientific articles on the comparative performance of various treatment modalities in order to arrive at evidence-based clinical decisions. This process requires the clinician to have a high level of expertise to appraise the design, methodology, analysis, and interpretation of clinical trials or systematic reviews in order to deduce valid conclusions. Such expertise needs to be provided by dental undergraduate or postgraduate educational curricula, in order to enable future dental clinicians to provide high quality evidence-based oral care.

The current Erasmus guidelines for postgraduate education in orthodontics in Europe^9^ indicate that after program completion, orthodontic postgraduates should, among other thing, be able to (i) apply the principles of evidence-based medicine, (ii) assess the quality of evidence and validity of conclusions, (iii) use electronic databases efficiently to obtain the evidence, (iv) understand and evaluate statistical methods and interpretation of findings in current literature, (v) perform an analytical review of research papers, (vi) apply data processing procedures, (vii) and interpret their own research findings.

Previous studies have assessed the knowledge of biostatistics among postgraduate students or residents in health sciences.^3,10,11,13,14,17,19^ Polychronopoulou et al^13^ found a moderate level of biostatistical knowledge among orthodontic postgraduate students, with a mean correct answer score of 44% and with only 12% of postgraduate students being able to correctly identify a commonly used chi-squared test. Best and Laskin^3^ made similar observations among oral and maxillofacial surgery residents, where the mean percentage of correct answers was 38%; additionally, 42% of them could not correctly identify continuous, ordinal, or nominal variables. Among dental postgraduate students from various specialties, Penmetsa et al^14^ found that only 15% of them could correctly interpret a p-value, and Sharma et al^16^ revealed that only 56% of them could correctly interpret a confidence interval. Similarly, moderate knowledge of biostatistics was identified for medical residents,^19^ where the mean knowledge score was 41%. American dental students assessed by Straub-Morarend et al^17^ were found to have moderate knowledge of basic evidence-based concepts (e.g. ranking of evidence, PubMed searches, meta-analysis, sensitivity/specificity, prevalence/incidence, etc) with correct answers ranging from 31.9% to 73.9% with a mean of 56%, which agrees with evidence of similarly insufficient knowledge among Finnish dental students.^11^

However, knowledge of biostatistics is only one prerequisite for evidence-based knowledge; critical appraisal, principles of epidemiology or research methodology are equally important. Therefore, the primary aim of this study was to assess the knowledge of postgraduate dental students from three different universities in Europe and the US on evidence-based research principles and research methodology. Secondarily, differences across different dental specialties were investigated.

## Materials and Methods

Ethical approval for this anonymous and voluntary survey was sought and waived from the appropriate committees of all three institutions. For Boston University and the University of Zürich, ethical clearance identifiers existed (Boston University: H-36703; University of Zürich: BASEC Request-Nr. Req-2017-0526), while for the University of Athens no formal identifier was generated, according to the University’s regulations. All participants verbally agreed to participate on their own initiative and agreed to the analysis and publication of the data.

The present article reports the results of a cross-sectional survey of senior (2nd, 3rd, or 4th year) postgraduate dental students from three academic institutions: Boston University; the University of Athens, and the University of Zürich. No sample size calculation was performed, and a convenience sample of all available eligible postgraduate students was taken. The questionnaire was printed for distribution and included demographics of the participants and 10 questions on evidence-based research or research methodology.

The questions covered:

1. Sample size calculation and its effect on the results/precision of a trial
2. Calculation of diagnostic positive predictive value of a diagnostic modality
3. Power of a study in relation to sample size and interpretation of the results
4. Appropriate method for random allocation sequence
5. Calculation of Number Needed to Treat (NNT) from relative/absolute risk reduction
6. Entities that can be blinded in a trial
7. PubMed MeSH term not relevant to a search for sensitivity/specificity of a diagnostic modality
8. Systematic review credibility related to the included studies
9. Identification of the proper diagram for addressing publication bias in meta-analysis
10. Diagrams used in randomised trials/systematic reviews (CONSORT flow diagram, forest plot, funnel plot, network plot)

Each question had a single correct answer, except the last question, which had four separate figures to be assigned to four answers. The 2nd and 5th questions required some simple calculations and provided free space for notes.

Data, including participant demographics and answers to each question, were extracted from the completed anonymous questionnaires independently by two authors (SNP, DK) to eliminate typographical and other errors. Apart from correct answer proportion on the question level, the overall correct answer percentage was calculated per participant by assigning one point to each question (0.25 points for each sub-part of the last question) and dividing the sum by 10. Normality of continuous variables (% correct survey answers) was checked and confirmed through visual inspection and formally with the Shapiro-Wilk test (p = 0.98). Descriptive statistics included means and SD for continuous variables and absolute/relative frequencies for categorical variables. Initial demographic differences across the three university samples were appropriately checked with one-way ANOVA, chi-squared test, or Fisher’s exact test. Predictors of the overall percent score for the whole questionnaire were checked with crude (univariable) linear regression and expressed as unstandardised regression coefficients (b) and the corresponding 95% confidence intervals (CI). An adjusted (multivariable) model was built to check differences in % overall score according to dental specialty by selecting confounders pertaining to at least 10% ‘change-in-estimate’.^8^ Differences in the correct answer percentages for each separate question according to student characteristics were checked with chi-squared tests, Fisher’s exact tests, or logistic regression. All analyses were run in Stata SE 14.0 (Stata; College Station, TX, USA) and the dataset was openly provided through Zenodo (http://doi.org/10.5281/zenodo.2631891).^12^

## Results

A total of 96 questionnaires were distributed to and completed by senior postgraduate students at the three participating universities: 39 from Boston University, 35 from the University of Athens, and 22 from the University of Zürich (Table 1). Each postgraduate student participated voluntarily in the survey and there were no dropouts. About half of the postgraduate students were male (47%; n = 45) and their average age was 30.0 years (SD 3.1 years), with statistically significant but clinically negligible differences among universities (average ages of 28.4, 30.6, and 31.6 for the universities in Athens, Boston, and Zürich, respectively; p < 0.001). An average of 5.0 years had elapsed since obtaining their DDS (Doctor of Dental Surgery) or DMD (Doctor of Dental Medicine) degree, 22% (n = 21) of the students had also obtained degrees other than DDS/DMD, 2% (only 2) had obtained a degree in epidemiology/biostatistics, and the vast majority of them (95%; n = 91) had taken an epidemiology/biostatistics course at their respective universities. The included sample of 96 students sought postgraduate programs in orthodontics (23.4%), prosthodontics (23.4%), endodontics (18.1%), paediatric dentistry (14.9%), periodontics (12.8%), and operative dentistry (7.5%).

**Table 1:** Characteristics of the included sample

	Total	Boston University	University of Athens	University of Zürich	p-value
n	96	39	35	22	
Age, mean (SD)	30.0 (3.1)	30.6 (3.0)	28.4 (2.3)	31.6 (3.4)	<0.001
Male, n (%)	45 (47%)	22 (56%)	13 (37%)	10 (45%)	0.25
Time since dental degree in years, mean (SD)	5.0 (2.4)	4.9 (3.1)	5.0 (1.5)	5.3 (2.3)	0.90
Also had degree other than dental, n (%)	21 (22%)	9 (23%)	7 (20%)	5 (23%)	0.94
Had epidemiology/statistics degree, n (%)	2 (2%)	2 (5%)	0 (0%)	0 (0%)	0.22
Had taken epidemiology/statistics course, n (%)	91 (95%)	38 (97%)	35 (100%)	18 (82%)	0.01

n = number of participants; SD=standard deviation. *Differences among the three universities checked with one-way ANOVA, chi-squared test, or Fisher’s exact test.

The overall correct answer score among the 96 postgraduate students was 45.6% (SD 15.0%) and ranged between 20.0% and 70.0%. No statistically significant differences were found in the overall % score according to the students’ characteristics in either the crude (Table 2) or adjusted analysis (Table 3), with the sole exception of dental specialty, for which paediatric dental postgraduate students had 16.4% significantly higher scores compared to periodontic postgraduate students (b = 15.1%; 95% CI = 3.0% to 27.1%; p = 0.02).

**Table 2:** Crude (univariable) linear regression analysis of the effect of various characteristics on overall % score

Factor*	Category	b	95% CI	p-value
Age	Per year	-0.5%	-1.5% to 0.5%	0.29
Sex	Female	Reference		
	Male	-1.4%	-7.5% to 4.7%	0.65
Time since dental degree	Per year	-0.9%	-2.2% to 0.4%	0.17
Also had degree other than dental	No	Reference		
	Yes	5.0%	-2.3% to 12.3%	0.18
University	Boston University	Reference		0.50†
	University of Athens	2.7%	-4.3% to 9.7%	
	University of Zürich	4.6%	-3.4% to 12.6%	
Dental specialty	Periodontics	Reference		0.08†
	Orthodontics	5.9%	-4.6% to 16.4%	
	Operative dentistry	1.4%	-12.5% to 15.3%	
	Endodontics	4.7%	-6.3% to 15.7%	
	Pedodontics	16.4%	4.9% to 27.9%	
	Prosthodontics	3.6%	-6.9% to 14.1%	
Had taken epidemiology/statistics course	No	Reference		
	Yes	-0.4%	-14.1% to 13.4%	0.96

b: unstandardised regression coefficient indicates mean difference in knowledge score between the level of the examined variable and the reference category. CI: confidence interval. Reference: the baseline category or level of the examined variable. *The variable ‘had epidemiology/statistics degree’ was not tested, as only two participants had such a degree. †p-value for overall Wald test.

**Table 3:** Adjusted (multivariable) linear regression analysis on the effect of dental specialty on overall % score to the survey, after adjusting for selected confounders from Appendix 1

Factor	Category	b	95% CI	p-value
Age	Per year	-0.7%	-2.1% to 0.7%	0.33
Time since dental degree	Per year	-0.2%	-1.9% to 1.4%	0.78
University	Boston University	Reference		0.12†
	University of Athens	3.0%	-4.9% to 10.9%	
	University of Zürich	9.9%	0.4% to 19.4%	
Dental specialty	Periodontics	Reference		0.05†
	Orthodontics	1.6%	-9.7% to 12.9%	
	Operative dentistry	1.7%	-13.6% to 17.0%	
	Endodontics	4.1%	-7.5% to 15.7%	
	Pedodontics	15.1%	3.0% to 27.1%	
	Prosthodontics	-2.4%	-13.9% to 9.2%	

b: unstandardised regression coefficient indicates mean difference in knowledge score between the level of the examined variable and the reference category. CI: confidence interval. Reference: the baseline category or level of the examined variable. *The variable ‘had epidemiology/statistics degree’ was not tested, as only two participants had such a degree. †p-value for overall Wald test.

**Fig 1:**
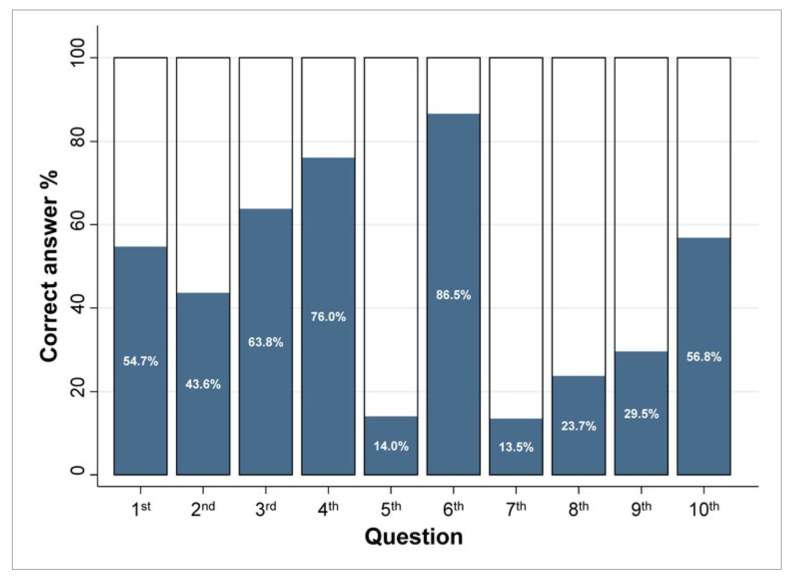
Bar plot of the correct answer percentage among the 96 postgraduate students for each separate question of the survey.

As far as each survey question is concerned, the percentage of the correctly answered questions ranged from 13.5% to 86.5% (Table 4; Fig 1). The questions with the poorest results pertained to the identification of PubMed keywords for sensitivity/specificity (13.5%), calculation of the NNT (14.0%), and identification of threats to the credibility of systematic reviews (23.7%). The questions most often correctly answered pertained to identification of entities that can be blinded in a trial (86.5%), identification of correct methods to generate a randomisation sequence (76.0%), and identification of the importance of a trial’s statistical power (63.8%).

**Table 4:** Answers to each question of the survey

Question related to	Correct n (%)	Incorrect n (%)
1. a priori sample size calculation	51 (54.7%)	44 (46.3%)
2. Calculation of diagnostic positive predictive value	41 (43.6%)	53 (56.4%)
3. Power of a study	60 (63.8%)	34 (36.2%)
4. Correct random allocation sequence	73 (76.0%)	23 (24.0%)
5. Number needed to treat	13 (14.0%)	80 (86.0%)
6. Blinding in trials	83 (86.5%)	13 (13.5%)
7. Sensitivity and specificity for diagnostic studies	13 (13.5%)	83 (86.5%)
8. Systematic review credibility	22 (23.7%)	71 (76.3%)
9. Diagram for publication bias	28 (29.5%)	67 (70.5%)
10. Diagrams in randomised trials / systematic reviews	54 (56.8%)	41 (43.2%)

## Discussion

This international survey indicates that the knowledge among the assessed 96 dental postgraduate students was moderate (mean score of 45.6%) and the percentages for correct answers ranged for each individual question from 13.5% to 86.5% (Table 4). This is to our knowledge the first study to solely assess the knowledge of evidence-based research methodology of dental postgraduate students. The relatively bleak picture seen in terms of knowledge among senior postgraduate dental students indicates that efforts should be reinforced in the systematic teaching of evidence-based research methodology in postgraduate dental curricula, but without contributing further to their well-documented stress and burnout.^5^ All participants in this survey had taken a biostatistics/epidemiology course as part of their postgraduate program. It is not clear whether all participants had attended a similar course in the undergraduate curriculum, as postgraduate students might come from different universities and might have received different basic dental education. The focus of this survey was on postgraduate curriculum education. The biostatistics/epidemiology course at each of the three universities concerned was structured on quite a similar basis. In essence, the content of the course involved introduction to study different designs, biostatistics (basic analytical approaches) and data processing methodology, descriptive and inferential types of data processing, basic reporting of data and interpretation of study findings.

Some comparisons can be made, however, with previous studies that assessed a combination of questions on biostatistics and research methodology. The proper method to generate a random number sequence for a randomised trial was answered correctly by approximately three quarters of the respondents, while a previous study identified a considerably lower percentage of correct answers, up to 48%.^13^ Entities that can be blinded in a triple-blind clinical trial were correctly identified by the majority of the participating students in this survey, while examples of double-blinding were correctly identified in 40% of postgraduate dental students according to Penmetsa et al,^14^ 69% of medical/dental postgraduate students in the study by Wadhwa et al,^18^ or 78% of orthodontic postgraduate students in the study by Polychronopoulou et al.^13^ Overall, compared to previous studies with similar questions on research methodology, it seems that the knowledge of postgraduate dental students included in this study tended to be better. However, the overall level of knowledge documented was moderate.

Similar findings have been reported about the limited knowledge of not only medical and dental students but also instructors about basic statistics.^1,6,7,10,11,17,19^ This lack of understanding in the context of research methodology may lead to both an erroneous interpretation of research findings as well as an inability to critically review the evidence presented in relevant articles. This raises a question about the potential for applicability of clinical research in practice and the necessity for authors of scientific articles to emphasise their findings in a clear and concise manner in the Results and Conclusions sections.

Moreover, an interesting finding of the present study was that negligible differences were detected in research methodology knowledge among the various dental specialties. The exception was paediatric dentistry students, who tended to outperform the rest in terms of average scores. The higher knowledge scores of paediatric dentistry students is noteworthy, although some uncertainty exists for this estimate considering the confidence limits. This could be attributed to specialisation-related parameters at some or all 3 universities participating in this survey, which may have influenced their ability to understand evidence-based research. Such parameters may include special short-term courses on research methodology given by the specialisation-program coordinator, or individual student-related competencies driven by personal interest and experience. No differences in knowledge level could be found among the responders in terms of demographic characteristics, including age, sex, time elapsed since dental degree, or having completed another, non-dentistry degree. Because only two students reported having attained an epidemiology/biostatistics degree, this was not formally tested in the univariable/multivariable regressions. Labrague et al^10^ reported that evidence-based knowledge was significantly higher for students with access to the internet and scientific journals. Some studies have reported sex differences in the biostatistics knowledge of medical residents or family physicians,^4,10,19^ but these seem to be isolated findings. Finally, students’ knowledge of evidence-based practice was not found to be closely related to their subjective assessment of their literature retrieval skills.^11^

The present study has also certain limitations. For instance, including only three universities (two from Europe and one from the US) provided only a small spectrum of participants and potentially limits the generalisability of the findings. Second, the participating postgraduate students were relatively unevenly distributed between universities and dental specialties. Third, the questionnaire was administered to senior postgraduate students (at least in their 2nd year) who had already received some prerequisite courses/training in clinical research methodology or epidemiology; previous studies indicated this to be a significant predictor.^13,19^ Pooling data from senior postgraduate students in their 2nd, 3rd, or 4th year should not introduce bias, since no trend in the biostatistics/clinical epidemiology skills of postgraduate students has been found.^2,15^ However, the findings might not be necessarily transferable to 1st year postgraduate dental students. Notwithstanding, it is the cumulative knowledge that is expected to be at the highest level and for which the educational curricula must strive; this is most likely represented by the senior students prior to program completion. Finally, the questionnaire used for this survey was tailored to this study, and has not been formally validated. However, face validity can be expected due to the authors’ previous experience with knowledge surveys and evidence-based methods.

The survey’s scope was to record and identify students’ knowledge at the postgraduate level, as well as to detect possible cross-specialisation differences in understanding of evidence-based research. We did not target the undergraduate curriculum, as this might have been advanced knowledge for an undergraduate student, whose primary focus is on core and basic aspects of dental science. It might have been interesting to administer the questionnaire to non-health science students, for example to students enrolled in mathematics or statistics courses. However, our primary aim was to detect the knowledge level of students enrolled in clinically-oriented postgraduate courses, as the target was not knowledge and understanding in statistics alone, but rather in clinical and research methodology overall. Future studies are planned to administer the questionnaire to certain dental-specialisation students at a greater number of universities in an attempt to achieve standardisation and calibration of the procedure.

## Conclusion

The mean percentage of correct answers given by postgraduate dental students to an questionnaire on evidence-based medicine and research methodology was 45.6%. This moderate score indicates that courses on evidence-based research methodology should be integrated in dental postgraduate curricula. This score was not influenced by age, gender, years elapsed since graduation, or other advanced degree; the sole parameter which seemed to influence this score was dental specialisation, where paediatric dentistry students tended to outperform the rest. The lack of knowledge of more than two-thirds of the responders about sensitivity/specificity, numbers needed to treat, and the credibility of clinical trials in systematic reviews was confirmed.

## Appendix

Variable selection for adjusted analyses. A model is constructed with the variable ‘dental specialty’ inserted. Then all variables are added one at a time and a minimal change-in-estimate of ±5% is used as selection criterion. This table describes the statistical procedure followed to identify predictors that are to be retained in the final multivariable model (Table 3).

**Table:**

Model	β	% change in β	Select
Basic model (specialty)	0.96	–	–
Basic + age	0.86	+10.4%	Yes
Basic + sex	0.95	+1.0%	No
Basic + time since dental degree	0.59	+38.5%	Yes
Basic + degree other than dental	0.92	+4.2%	No
Basic + university	0.84	12.5%	Yes
Basic + had relevant course	0.96	0%	No

β: regression coefficient. Indicates mean difference in knowledge score between the level of the examined variable and the reference category.
